# Nasal Polypi Cured with One Dose of Aurum-M,^50m^

**Published:** 1892-12

**Authors:** J. R. Hayes

**Affiliations:** Indianapolis, Ind.


### NASAL POLYPI CURED WITH ONE DOSE OF AURUM-M,$^{50M}$.

J. R. HAYNES, M. D., INDIANAPOLIS, IND.

Mr. H. T., aged about seventy-eight years, rather stout but not very fleshy, would weigh about one hundred and sixty, hair and beard milk white. Some forty years ago had nasal polypi of both anterior nares. Had them extracted by some "regular," and was thoroughly dosed by him. In a short time they grew again, but larger than before; he had the same M.D. extract them the second time; and as he reported, gave him any amount of medicine as well as used external washes with astringent nasal injections, but the polypi persisted in growing for the third time, when he refused to have them extracted again. They filled both anterior nares, so much so, that it was impossible for him to force a particle of air through the nasal passages, and protruded so as to come down to near the border of the upper lip; was very sensitive to the touch; of a grayish reddish color, and a continuous semi-putrid discharge which was very disagreeable to him and every one that he came in contact with.

He was very despondent, said he was nothing but a nuisance and had been for the past thirty years (after the third growth of the polypi), and that the sooner he was dead and out of the way the better for all concerned, as he could see no benefit to himself or any one else by his doing so; after getting his symptoms the best that I could (placing the most importance upon the mental), and mistrusting sycotic basis, I gave him one small dose of Aurum$^{50m}$, to be followed by Sac-lac. once in forty-eight hours.

In about three months both polypi entirely disappeared and have shown no symptoms of returning for the past two years.

### DISCUSSION.

Dr. E. E. Case—About a year and a half ago I had a case of polypus in a lady. She was a fleshy person, a Calcarea subject, and she received that remedy in the 1m potency. The polypus was so large that the nostrils were entirely occluded, even the face was perceptibly swollen. Under treatment this swelling decreased, the offensive discharges lessened, and she began to have a bleeding from the nose, the polypus in the meanwhile growing smaller. Some of her friends concluded, notwithstanding the improvement, that there was no use in doctoring, and she did not come back. Six months after the prescription, when blowing her nose, the remnants of the polypus came out on her handkerchief.

Dr. Baylies—I also cured a polypus in a child eight years old with a single dose of Calc-carb.$^{m}$ (million) F.

